# Effect of prohylactic mesh in elective laparotomy

**DOI:** 10.6026/973206300212704

**Published:** 2025-08-31

**Authors:** Rohit Dubey, Nishi Mishra, Nitish Adawadkar, Shobhit Gupta, Tanya Jonwal

**Affiliations:** 1 Department of Surgery, Government Medical College Satna, Madhya Pradesh, India; 2 Department of Obstetrics & Gynaecology, Government Medical College Satna, Madhya Pradesh, India; 3 Department Of Community Medicine Government Medical College Ratlam Madhya Pradesh India; 4 Department of Medicine F.H Medical College Agra, Uttar Pradesh, India; 5 Department of Community Medicine Government Medical College Ratlam Madhya Pradesh India

**Keywords:** Incisional hernia, obesity, laparotomy

## Abstract

A hernia is a defect in the fascia of the abdominal wall and thus resulting into the formation of a hernial sac of peritoneum that contains visceral organ or abdominal contents or other bulges that may appear similar, but are not true hernias. Incisional hernia is the bulging out of contents of abdominal cavity through a previous surgical defect in anterior abdominal wall and is a significant complication for patients who have undergone elective laparotomy. The risk of developing this condition increases substantially in individuals with risk factors such as obesity and chronic respiratory ailments. Various methods of suture closure and mesh reinforcement have been used to restore abdominal wall integrity and prophylactic treatment of incisional hernia. Failure of effective and sufficient closure of the abdominal wall after operations leaves the patient at risk for developing hernia. The risk of developing this condition increases substantially in individuals with risk factors such as obesity and chronic respiratory ailments. Therefore, it is of interest to evaluate the efficacy of preventive mesh placement following laparotomy.

## Background:

A hernia is a defect in the fascia of the abdominal wall and thus resulting into the formation of a hernia sac of peritoneum that contains visceral organ or abdominal contents or other bulges that may appear similar, but are not true hernias [1]. The incidence of incisional hernia develops after abdominal wall closure range widely from 10 to 23% and up to 69% in long-term high-risk patients [2]. Different techniques for suture closure (including material and method) and mesh reinforcement (considering position and shape) have been employed to restore the integrity of the abdominal wall and to provide preventive treatment for incisional hernia [3]. Despite advances in early repair, recurrence rates remain unacceptable (12-54%). Larger defect (>2-3 cms) shows higher recurrence rate around 10-15% if closed by primary repair [2]. Recurrence is susceptible to a vicious cycle of morbidity, because early subsequent repair presents greater technical challenges and an increased risk for recurrence and morbidity [4]. The inadequate and ineffective closure of the abdominal wall following surgical procedures places the patient at an increased risk of developing a hernia [5-6]. Although prophylactic reinforcement with mesh has been shown to reduce the risk of wound dehiscense and incisional hernia reinforcement of the suture line with a mesh may be an effective way of preventing wound dehiscence [7]. Therefore, it is of interest to show safety and efficacy of prohylactic mesh placement (PMP) in elective midline laparotomy.

## Methodology:

The study was carried out in 50 patients admitted in Department of General Surgery at M.Y. Hospital, Indore. The study was conducted after the clearance from Institutional Ethical Committee. An informed consent was taken from each patient after which the patient was taken for an elective surgery (prophylactic mesh replacement). Patients with age less than 18 years and with known comorbidities such as essential hypertension, diabetes, thyroid disease, renal disease, or any other medical illness, were excluded from the study. A comprehensive history encompassing personal details, medical history and medication usage was obtained with the help of questionnaire. The follow up was done for 1year post-surgery. After data collection; the data was entered into Microsoft Office Excel and analyzed using EpiInfo 7, free software.

## Results:

There were 8 (16.0%) patients in the age group 18-20 years, 27 (54.0%) patients in the age group 21-40 years, 12 (24.0%) patients were in the age group 41-60 years and 3 (6.0%) patients were in the age group >60 years. The mean age of the patients in study group was 37.60 ± 13.51 years with a range between 18 years to 70 years. In 5 (10%) patients onlay mesh was applied and in 45 (90.0%) patient's sublay mesh was applied. In majority of the patients sublay mesh was applied. In patients who were operated using onlay mesh, 3 (60.0%) patients had occurrence of hernia and in 2 (40.0%) patients there was no hernia. In patients who had undergone surgery using onlay mesh, higher prevalence of hernia was seen. The association between location of mesh and the final outcome was found to be statistically significant (p=0.001), showing that the final outcome is dependent on the location of mesh of the patients. The sublay technique of mesh placement was better in prophylactic prevention in incisional hernia development after elective laparotomies as shown in Table 1 (see PDF). In onlay mesh placed cases, all five patients had either of the wound complications, that is, SSI, seroma, or wound dehiscence. These complications were related to the prolonged hospital stay in all patients with onlay mesh. One out of five (20%) patients had stayed in ward up to 21 days and other four (80%) patients had stayed for more than 21 days. Three out of four (75%) patients who developed incisional hernia belonged to the onlay mesh placed group. The high rate of hernia in this group hence can be attributed to higher rate of wound complication in these patients.

## Discussion:

All 4 patients who developed hernia belonged to obese category as per WHO criteria for BMI classification. 4 out of 24 (16.7%) patient who were obese were associated with this complication. This was however not found to be clinically significant but we couldn't clearly rule out obesity as a risk factor for development of incisional hernia. One patient who developed hernia in sublay group had prolonged hospital stay due to development of complications (wound dehiscence). This proves that the sublay mesh placement as such is not associated with increased risk but the wound infection associated with the mesh leads to the hernia formation. In a study by Borab *et al.* [8] comparing the suture closure and onlay mesh placement, they found lower occurence of incisional hernia with mesh but seroma formation and other wound complications were present. This was consistent with our findings. As per the findings of Nachiappan *et al.* & Abbas *et al.* there is a significant reduction in incidence of incisional hernia when prophylactic mesh is applied [9, 10]. As per findings of Berta *et al.* the incidence of incisional hernia was high in patients undergoing midline laparotomy [11].

## Conclusion:

The mesh placement after elective laparotomy is associated with low occurrence of incisional hernia as compared with the primary suture closure. Onlay mesh placement technique has more chances of complications such as seroma and flap infections than sublay due to the fact of more dissection in fatty plane for mesh placement. Furthermore, more superficial location of mesh is easily accessible for bacterial invasion.

## Limitations:

The study is limited by the fact that many of the factors directly involved in wound healing like diabetic status, hemoglobin status of patient, protein status and technical aspect of closure. Also our study didn't comment on the deviations with normal suture closure of the abdominal cavity and the type of mesh used.

## References

[1] Llaguna O.H (2011). World J Surg..

[2] Bhangu A (2013). Hernia..

[3] El-Khadrawy OH (2009). Hernia..

[4] de Goede B (2015). Surgery..

[5] Jairam AP (2017). Lancet..

[6] Argudo N (2017). Cir Esp..

[7] Payne R (2017). Hernia..

[8] Borab ZM (2017). Surgery..

[9] Nachiappan S (2013). World J Surg..

[10] Abbas AW (2025). Hernia..

[11] Fabregó B (2024). Int J Gynecol Cancer..

